# Epidemiology of stroke in Northern Italy: the Cerebrovascular Aosta Registry, 2004–2008

**DOI:** 10.1007/s10072-012-1185-8

**Published:** 2012-09-25

**Authors:** Giovanni Corso, Edo Bottacchi, Guido Giardini, Marco Di Giovanni, Teodoro Meloni, Massimo Pesenti Campagnoni, Massimo Veronese Morosini

**Affiliations:** 1 Stroke Unit, Department of Neurology, Ospedale Regionale, Viale Ginevra n 3, 11100 Aosta, Italy; 2 Department of Radiology, Ospedale Regionale, Aosta, Italy; 3 Department of Emergency Room, Ospedale Regionale, Aosta, Italy; 4 Statistics Department, Ospedale Regionale, Aosta, Italy

**Keywords:** First-ever stroke, Ischaemic stroke subtypes, Population-based study, Case-fatality, Long-term functional outcomes

## Abstract

Our aim was to prospectively ascertain the incidence of first-ever stroke and ischaemic stroke subtypes, mortality, functional outcome and recurrence in Northern Italy. We identified all possible cases of stroke (1st January 2004 and 31st December 2008). Multiple overlapping sources were used. Standard definitions for incident cases, pathological types and infarction subtypes were used. Patient characteristics were identified and analysed, case-fatality was ascertained from administrative databases, and outcome was assessed in all surviving patients by modified Rankin Scale. We identified 1,326 incident strokes. The pathological diagnosis was confirmed in 94 % of cases. The incidence of first-ever stroke was 80.2 per 100,000 (95 % CI 73–87) when adjusted to world population. The incidence of embolic stroke was significantly greater in women than in men (*p* < 0.001) whereas the incidence of atherothrombotic stroke was significantly greater in men than in women (*p* < 0.001). The case-fatality of incident strokes was 9.5 % at 7 day, 16.1 % at 28 day, and 29.9 % at 1 year. Case-fatality of ischaemic stroke was lower than that of other pathological types (*p* < 0.0001). Hypertension was the most important risk factor, and atrial fibrillation was the most common in embolic stroke. Increasing age, female gender and embolic stroke subtypes were associated with an adverse outcome. Data on stroke incidence and case-fatality were similar to those of other high-income countries. However, differences were found in the distribution of risk factors and prognosis across the stroke types and ischaemic stroke subtypes. Gender differences in long-term functional outcomes were significant.

## Introduction

In the last decades, deaths from stroke have declined [1]. Despite the decline in mortality, the annual number of cases of stroke is expected to increase within the next few decades, mainly due to a 30 % growth in the elderly population [2].

Stroke is a heterogeneous disorder consisting of three major pathological types: ischaemic stroke, primary intracerebral haemorrhage, subarachnoid haemorrhage. Each type of stroke has distinct causes, different incidences and natural histories [3, 4]. Previous population-based studies of stroke have often focused on stroke types and ethnic groups [5–8], very little data exist regarding functional outcome [9–11]. Comparison of functional outcome and survival for each type of stroke and for all specific ischaemic subtypes could allow clinicians to identify patients who have the worst prognosis.

We report the incidence of first-ever stroke, case-fatality, previously known vascular risk factors, functional outcome and recurrence from the Cerebrovascular Aosta Registry (CARe), a large population-based prospective stroke incidence study undertaken in Aosta Valley (AV), Italy from 2004 to 2008.

## Methods

Methods of the CARe have been described elsewhere [12]. Aosta Valley is situated in the extreme North-West of Italy. It is a geographically isolated region with only one main town, Aosta (one-third of the population), and 73 municipalities. Data on vital status were obtained from the “Ufficio Anagrafe” which creates a unique personal identification number issued to all Aosta Valley residents and contains complete electronic follow-up data on civil registry number, name, gender, date and place of birth, vital status, address and emigration. The population was 123,748 inhabitants in 2004 and 125,366 inhabitants in 2008. Ninety-nine percent of the resident population is Caucasian, and 51 % is female. We prospectively checked all cases from overlapping sources: (1) daily review of computer-generated Emergency Department admission registry, (2) GP referrals, (3) daily review of neurological referrals and referrals to the Radiology Department, (4) monthly check of intensive-care unit, (5) monthly review of hospital diagnostic codes 430–438 according to the International Classification of Diseases, Ninth Revision (ICD9-CM) [13] and (6) monthly review of the mortality list of the Office of Legal Medicine (diagnostic codes 430–438). The ascertainment cases were obtained between 1st January 2004 and 31st December 2008, follow-up continued until 31st August 2011. The events detected were notified to the Study Group (GC, EB, GG) on a standard form. Patients notified were examined as soon as possible by one of three field investigators. Radiological data were reviewed by a radiologist (TM) who was aware of the clinical signs and symptoms. An investigator (GC) reviewed the medical records of all patients identified through sources. The Study Group categorised the patients by case, pathological stroke type, infarct subtype and cause according to the following definitions.

Stroke was defined as “rapidly developing focal (or global) clinical signs of cerebral dysfunction that lasted more than 24 hours (unless interrupted by death), with no apparent cause other than of vascular origin” [14]. Cerebral infarction, intracerebral haemorrhage and subarachnoid haemorrhage are based on standard definitions [15]. Stroke aetiology was classified prospectively according to the TOAST criteria [16]. Stroke subtype was assigned to each patient by two neurologists and in case of discrepancy, the patient records were reviewed. Cases were defined as individuals who had had a stroke and who were permanent residents in AV. The cases were classified as first-ever stroke, recurrent stroke and transient ischemic event (TIA) based on the clinical history. Stroke was classified as either first-ever: occurring for the first time in a patient’s life, or recurrent: defined though anamnestic recall, hospital records. A stroke in a patient with a previous TIA was coded as an incident. Patients who fulfilled the WHO criteria for stroke but for whom neither brain imaging nor autopsy first CT-scan had been performed >14 days after the event were classified as having undefined stroke [17]. When a patient died very rapidly all the available information coming from relatives, GPs, death certificates and hospital records was used. The GPs were invited by call or e-mail every 6 months to supply any information about patients with possible acute cerebrovascular events. All the patients notified by GPs were evaluated in the Outpatient Neurology Clinic. All surviving cases were regularly followed up by neurologists in the hospital clinic, by telephone interview or contact with close relatives, and new events were recorded. Onset of stroke symptoms was considered the starting point for follow-up. At the follow-up dependency was assessed by modified Rankin scale (mRS) [18]. As for mortality, the list of all patients included in CARe was compared every year with the official local mortality data. Every death, and death certificate, and the corresponding personal information in the computerised population register was crosschecked.

### Ethics

The study was approved by the Ethical Committee of Aosta Valley.

### Data analysis

Stroke incidence and 7-day, 28-day and 1 year case-fatality were analysed for the whole study period, by stroke pathological type and ischaemic stroke subtypes. We calculated incidence rates using the sum of the residential population as the denominator. Crude incidence rates per 100,000 population per year with corresponding 95 % confidence intervals (95 % CIs) were calculated by the use of standard approaches [19]. All incidence rates of first-ever stroke were adjusted to the standard 2001 Italian population [20], and WHO world population [21] using the direct method. Differences in patient characteristics, pre-morbid risk factors, and hospital investigations were assessed by χ^2^ test (for categorical variables) or analysis of variance (for continuous variables). The data and tests were considered as significant when the *p* value was ≤0.05.

Seven-day, 28-day, and 1-year case-fatality was defined as the proportion of cases for which death occurred within 7 days, 28 days, and 1 year of stroke onset, respectively.

We divided the patients in two categories (mRS 0–2, and mRS 3–5). Functionally dependent was defined as an mRS score of 3, 4, 5.

The Kaplan–Maier method was used to estimate rates of recurrent stroke after first cerebral infarction.

## Results

In total 2,825 patients with “possible events” were initially considered for the study. After careful review of the medical records by the expert panel 544 patients were excluded. In particular, 225 patients were not local residents, 62 for general deterioration/miscellaneous neurological disease, 10 for seizures, 25 for trauma, 143 for other clinical conditions (i.e. vertigo, metabolic disorders, intoxication, sepsis, falls, etc.), 71 because CT-scan detected an infarct or haemorrhage but there was no clinical expression of stroke and 8 for cerebral venous thrombosis.

Throughout the study period 1,326 patients were judged to have had a first-ever stroke, 435 were diagnosed as TIA (of whom 24 had a previous stroke) and 520 had a recurrent stroke. All patients were detected through active surveillance: 1,188 (89.6 %) cases were identified during their admission in the ER or hospitalisation (12 cases identified in the ER refused admission and 26 had a stroke while in hospital for other reasons); 94 (7.1 %) cases were notified by GPs (40 were hospitalised outside the region and 54 were subsequently evaluated in the neurology outpatient clinics); 44 (3.3 %) cases were identified through death certificates (for 4 of those identified through death certificates, we were able to obtain clinical and imaging information, and 40 died at home or at nursing home and the diagnosis was based on the clinical judgement of the GP). The hospital admission rate was 92.5 % (1,227/1,326). Patients were classified as ischaemic stroke in 1,057 (79.7 %) cases, as primary intracerebral haemorrhage in 146 (11 %) cases, as subarachnoid haemorrhage in 44 (3.3 %) cases and as undefined stroke in 79 (6 %) cases. 1,241 (93 %) patients had brain CT within 14 days of symptom onset; in one patient with subarachnoid haemorrhage the diagnosis was made by lumbar puncture; and in five cases the diagnosis was made at autopsy. The classification was made according to standard definitions and results of neuroimaging, necroscopy or lumbar puncture (for subarachnoid haemorrhage only) in 94 % of cases. The reasons for not doing CT were rapid death [30] or refusal by the patient (or the family) to be taken to the ER [21] late neurological consultation [28].

The majority of ischaemic strokes 1,028 (97.2 %) were managed in hospital. The median time from symptom onset to CT-scan was 5.4 h (IQR 3–15). Out of 1,057 patients 523 (49.5 %) had a brain MRI within the first week from stroke. The following investigations were carried out to determine causal subtypes of ischaemic stroke: carotid Doppler ultrasonography or transcranial Doppler ultrasonography in 844 (80 %) cases; magnetic resonance angiography or CT-scan angiography in 209 (19.7 %); cerebral angiography in 15 (1.4 %); electrocardiography in 1,048 (99 %); holter ECG in 151 (14.3 %); transthoracic echocardiography in 184 (17.4 %); transoesophageal echocardiography in 52 (5 %). The causes of cardiac sources of emboli were atrial fibrillation (206, 76 %) cardiomyopathy (22, 8 %), interatrial septal abnormalities (13, 5 %), acute myocardial infarction (15, 5 %), valvular heart disease (9, 3 %), chronic sinoatrial disorder (5, 2 %) and infectious endocarditis (2, 1 %). Of 1,057 cases of ischaemic stroke, 153 (14 %) were atherothrombotic; 266 (25 %) embolic; 210 (20 %) lacunar; 16 (2 %) were caused by other determined causes; and in 412 (39 %) the exact cause of ischaemic stroke remained unknown (undetermined).

The crude incidence of first-ever stroke was 212 per 100,000 population (95 % CI 200–223), and 80.2 per 100,000 (95 % CI 73–87) when adjusted to world populations (Table 1). Table 2 shows the age and sex-specific incidence rates by pathological types. Stroke incidence increased with increasing age, in women and men, for all stroke pathological types, except for subarachnoid haemorrhage. The age-adjusted incidence rates regarding ischaemic stroke subtypes are shown in Table 3. The incidence of embolic stroke was significantly greater in women than in men (*p* < 0.001) whereas the incidence of atherothrombotic stroke was significantly greater in men than in women (*p* < 0.001). Men had one and a half times higher risk of atherothrombotic stroke (*p* = 0.01), and women had one and a half times higher risk of embolic stroke (*p* = 0.002).

**Table 1:** Incidence rates per 100,000 population of first-ever stroke, by age and sex in Aosta Valley, Italy in 2004–2008

	Men			Women			Total		
	Number at risk	Number of cases	Incidence rate (95 % CI)	Number at risk	Number of cases	Incidence rate (95 % CI)	Number at risk	Number of cases	Incidence rate (95 % CI)
Age group (years)									
0–44	33,745	34	20.2 (13.4–26.9)	31,869	19	11.9 (6.6–17.3)	65,614	53	16.2 (11.8–20.5)
45–54	9,115	38	83.4 (56.9–109.9)	8,777	21	47.9 (27.4–68.3)	17,892	59	66 (49.1–82.8)
55–64	7,933	97	244.5 (195.9–293.2)	7,700	38	98.7 (67.3–130.1)	15,633	135	172.7 (143.6–201.8)
65–74	6,341	177	558.3 (476.3–640.3)	7,171	123	343 (282.5–403.6)	13,512	300	444 (393.9–494.2)
75–84	3,623	220	1,214.5 (1,055–1,374)	5,670	263	927.7 (816.1–1,039.3)	9,293	483	1,039.5 (947.3–1,131.7)
≥85	831	85	2,045.7 (1,615.3–2,476.2)	2,328	211	1,812.7 (1,570.3–2,055.1)	3,159	296	1,874 (1,662.5–2,085.5)
All	61,588	651	211.4 (195.2–227.6)	63,515	675	212.5 (196.5–228.6)	125,103	1,326	212 (200.6–223.4)
Standardised^a^			228.7 (211.9–245.6)			157 (143.2–170.8)			189.3 (178.5–200)
Standardised^b^			100 (88.8–111.2)			62.1 (53.4–70.8)			80.2 (73.2–87.2)

^a^Age-adjusted rate standardised to the Italian population

^b^Age-adjusted rate standardised to the world population

**Table 2:** Pathological stroke types incidence per 100,000 by type, sex, and age in Aosta Valley, Italy in 2004–2008

	Number at risk	Ischaemic stroke		Primary intracerebral haemorrhage		Subarachnoid haemorrhage		Undefined stroke subtype	
		*n*	Incidence rate (95 % CI)	*n*	Incidence rate (95 % CI)	*n*	Incidence rate (95 % CI)	*n*	Incidence rate (95 % CI)
Men									
0–44	33,745	20	11.9 (6.7–17)	4	2.4 (0–4.7)	9	5.3 (1.8–8.8)	1	0.6 (0–1.8)
45–54	9,115	26	57 (35.1–79)	8	17.6 (5.4–29.7)	2	4.4 (0–10.5)	2	4.4 (0–10.5)
55–64	7,933	76	191.6 (148.6–234.6)	12	30.3 (13.1–47.4)	6	15.1 (3–27.2)	3	7.6 (0–16.1)
65–74	6,341	149	470 (394.7–545.2)	19	59.9 (33–86.9)	2	6.3 (0–15)	7	22.1 (5.7–38.4)
75–84	3,623	175	966 (823.6–1,108.5)	31	171.1 (110.9–231.3)	3	16.6 (0–35.3)	11	60.7 (24.8–96.6)
≥85	831	67	1,612.5 (1,229.5–1,995.5)	10	240.7 (91.7–389.7)	0	0 (0–0)	8	192.5 (59.2–325.8)
Subtotal	61,588	513	166.6 (152.2–181)	84	27.3 (21.4–33.1)	22	7.1 (4.2–10.1)	32	10.4 (6.8–14)
Standardised^a^			180.4 (165.4–195.4)		29.3 (23.2–35.3)		7.1 (4.1–10)		12 (8.2–15.9)
Standardised^b^			41.1 (33.9–48.2)		8.4 (5.1–11.6)		5.7 (3–8.4)		2.3 (0.6–4)
Women									
0–44	31,869	12	7.5 (3.3–11.8)	3	1.9 (0–4)	2	1.3 (0–3)	2	1.3 (0–3)
45–54	8,777	11	25.1 (10.3–39.9)	2	4.6 (0–10.9)	6	13.7 (2.7–24.6)	2	4.6 (0–10.9)
55–64	7,700	29	75.3 (47.9–102.7)	1	2.6 (0–7.7)	5	13 (1.6–24.4)	3	7.8 (0–16.6)
65–74	7,171	105	292.8 (236.9–348.8)	9	25.1 (8.7–41.5)	4	11.2 (0.2–22.1)	5	13.9 (1.7–26.2)
75–84	5,670	220	776 (673.9–878.2)	27	95.2 (59.3–131.1)	2	7.1 (0–16.8)	14	49.4 (23.5–75.2)
≥85	2,328	167	1,434.7 (1,218.7–1,650.7)	20	171.8 (96.6–247.1)	3	25.8 (0–54.9)	21	180.4 (103.3–257.5)
Subtotal	63,515	544	171.3 (156.9–185.7)	62	19.5 (14.7–24.4)	22	6.9 (4–9.8)	47	14.8 (10.6–19)
Standardised^a^			125.9 (113.6–138.3)		14.2 (10–18.3)		6.2 (3.5–9)		10.7 (7.1–14.2)
Standardised^b^			47.8 (40.2–55.4)		5.6 (3–8.3)		4.3 (2–6.5)		4.4 (2.1–6.7)
Total									
0–44	65,614	32	9.8 (6.4–13.1)	7	2.1 (0.6–3.7)	11	3.4 (1.4–5.3)	3	0.9 (0–1.9)
45–54	17,892	37	41.4 (28–54.7)	10	11.2 (4.2–18.1)	8	8.9 (2.7–15.1)	4	4.5 (0.1–8.9)
55–64	15,633	105	134.3 (108.7–160)	13	16.6 (7.6–25.7)	11	14.1 (5.8–22.4)	6	7.7 (1.5–13.8)
65–74	13,512	254	376 (329.8–422.1)	28	41.4 (26.1–56.8)	6	8.9 (1.8–16)	12	17.8 (7.7–27.8)
75–84	9,293	395	850.1 (766.6–933.6)	58	124.8 (92.7–156.9)	5	10.8 (1.3–20.2)	25	53.8 (32.7–74.9)
≥85	3,159	234	1,481.5 (1,293.1–1,669.9)	30	189.9 (122–257.8)	3	19 (0–40.5)	29	183.6 (116.8–250.4)
Subtotal	125,103	1,057	169 (158.8–179.2)	146	23.3 (19.6–27.1)	44	7 (5–9.1)	79	12.6 (9.8–15.4)
Standardised^a^			150.6 (141–160.2)		20.8 (17.2–24.4)		6.7 (4.7–8.8)		11.2 (8.6–13.8)
Standardised^b^			61.5 (55.3–67.6)		9 (6.7–11.4)		5.3 (3.5–7.1)		4.4 (2.8–6)

^a^Age-adjusted rate standardised to the Italian population

^b^Age-adjusted rate standardised to the world population

**Table 3:** Incidence rates per 100,000 per year for different subtypes of ischaemic stroke by age and sex in Aosta Valley, Italy in 2004–2008

Number at risk		Atherothrombotic		Embolic		Lacunar		Other		Undetermined	
		*n*	Incidence rate (95 % CI)	*n*	Incidence rate (95 % CI)	*n*	Incidence rate (95 % CI)	*n*	Incidence rate (95 % CI)	*n*	Incidence rate (95 % CI)
Men											
0–44	33,745	0	0 (0–0)	4	2.4 (0–4.7)	0	0 (0–0)	5	3 (0.4–5.6)	11	6.5 (2.7–10.4)
45–54	9,115	1	2.2 (0–6.5)	5	11 (1.4–20.6)	7	15.4 (4–26.7)	4	8.8 (0.2–17.4)	9	19.7 (6.8–32.6)
55–64	7,933	13	32.8 (15–50.6)	8	20.2 (6.2–34.1)	31	78.2 (50.7–105.7)	0	0 (0–0)	24	60.5 (36.3–84.7)
65–74	6,341	28	88.3 (55.6–121)	29	91.5 (58.2–124.7)	34	107.2 (71.2–143.3)	2	6.3 (0–15)	56	176.6 (130.4–222.8)
75–84	3,623	37	204.2 (138.5–270)	39	215.3 (147.8–282.8)	30	165.6 (106.4–224.8)	0	0 (0–0)	69	380.9 (291.2–470.6)
≥85	831	12	288.8 (125.6–452)	21	505.4 (289.8–721)	14	336.9 (160.7–513.1)	0	0 (0–0)	20	481.3 (270.9–691.8)
Subtotal	61,588	91	29.6 (23.5–35.6)	106	34.4 (27.9–41)	116	37.7 (30.8–44.5)	11	3.6 (1.5–5.7)	189	61.4 (52.6–70.1)
Standardised^a^			32.2 (25.9–38.6)		38.9 (32–45.9)		40 (32.9–47.1)		3.5 (1.4–5.6)		65.7 (56.7–74.8)
Standardised^b^			11.8 (8–15.6)		14.9 (10.6–19.2)		17.5 (12.8–22.1)		3.5 (1.4–5.6)		28.8 (22.8–34.8)
Women											
0–44	31,869	0	0 (0–0)	0	0 (0–0)	0	0 (0–0)	1	0.6 (0–1.9)	11	6.9 (2.8–11)
45–54	8,777	0	0 (0–0)	3	6.8 (0–14.6)	1	2.3 (0–6.7)	1	2.3 (0–6.7)	6	13.7 (2.7–24.6)
55–64	7,700	2	5.2 (0–12.4)	6	15.6 (3.1–28.1)	5	13 (1.6–24.4)	2	5.2 (0–12.4)	14	36.4 (17.3–55.4)
65–74	7,171	11	30.7 (12.6–48.8)	26	72.5 (44.6–100.4)	21	58.6 (33.5–83.6)	1	2.8 (0–8.3)	46	128.3 (91.2–165.3)
75–84	5,670	30	105.8 (68–143.7)	55	194 (142.8–245.2)	45	158.7 (112.4–205.1)	0	0 (0–0)	90	317.5 (252–382.9)
≥85	2,328	19	163.2 (89.9–236.6)	70	601.4 (460.9–741.8)	22	189 (110.1–267.9)	0	0 (0–0)	56	481.1 (355.4–606.8)
Subtotal	63,515	62	19.5 (14.7–24.4)	160	50.4 (42.6–58.2)	94	29.6 (23.6–35.6)	5	1.6 (0.2–3)	223	70.2 (61–79.4)
Standardised^a^			13.9 (9.8–18)		35.3 (28.8–41.9)		21.8 (16.7–27)		1.6 (0.2–2.9)		53.4 (45.3–61.4)
Standardised^b^			4.4 (2.1–6.6)		11.5 (7.8–15.3)		7.5 (4.5–10.6)		1.3 (0–2.5)		23.1 (17.8–28.4)
Total											
0–44	65,614	0	0 (0–0)	4	1.2 (0–2.4)	0	0 (0–0)	6	1.8 (0.4–3.3)	22	6.7 (3.9–9.5)
45–54	17,892	1	1.1 (0–3.3)	8	8.9 (2.7–15.1)	8	8.9 (2.7–15.1)	5	5.6 (0.7–10.5)	15	16.8 (8.3–25.3)
55–64	15,633	15	19.2 (9.5–28.9)	14	17.9 (8.5–27.3)	36	46.1 (31–61.1)	2	2.6 (0–6.1)	38	48.6 (33.2–64.1)
65–74	13,512	39	57.7 (39.6–75.8)	55	81.4 (59.9–102.9)	55	81.4 (59.9–102.9)	3	4.4 (0–9.5)	102	151 (121.7–180.3)
75–84	9,293	67	144.2 (109.7–178.7)	94	202.3 (161.4–243.2)	75	161.4 (124.9–197.9)	0	0 (0–0)	159	342.2 (289.1–395.3)
≥85	3,159	31	196.3 (127.2–265.3)	91	576.1 (458.1–694.2)	36	227.9 (153.6–302.3)	0	0 (0–0)	76	481.2 (373.2–589.1)
Subtotal	125,103	153	24.5 (20.6–28.3)	266	42.5 (37.4–47.6)	210	33.6 (29–38.1)	16	2.6 (1.3–3.8)	412	65.9 (59.5–72.2)
Standardised^a^			21.6 (17.9–25.2)		37.4 (32.7–42.2)		30 (25.7–34.3)		2.5 (1.3–3.8)		59 (53–65)
Standardised^b^			7.7 (5.5–9.9)		13.3 (10.4–16.2)		12.3 (9.6–15)		2.4 (1.2–3.6)		25.8 (21.8–29.8)

^a^Age-adjusted rate standardised to the Italian population

^b^Age-adjusted rate standardised to the world population

The frequencies of risk factors are shown in Table 4. History of hypertension was highly prevalent in all groups and hypertension was significantly more prevalent in primary intracerebral haemorrhage (*p* = 0.005). In addition, 20 % had a known previous atrial fibrillation and 17 % of patients were diabetic. Twenty-two percent of patients (235/1,057) had atrial fibrillation before their ischaemic stroke: 9 % of male patients and 13 % of female patients. The average age in the atrial fibrillation group at the time of ischaemic stroke was 83 years for women and 77 years for men (*p* < 0.0001).

**Table 4:** Risk factors among patients with first-ever stroke by pathological type and ischaemic stroke subtype in CARe Aosta Valley, Italy 2004–2008

		First-ever stroke		Ischaemic stroke												Primary intracerebral haemorrhage		Subarachnoid haemorrhage		p*
		Total (*n* = 1,326)		Total (*n* = 1,057)		Atherothrombotic (*n* = 1,530		Embolic (*n* = 266)		Lacunar (*n* = 210)		Other (*n* = 16)		Undetermined (*n* = 412		(9*n* = 146)		(*n* = 44)		
Baseline characteristic																				
Mean (SD) age (years)		75 (13.7)		75.7 (12.7)		77.8 (9.2)		79.2 (11.3)		74.7 (10.9)		47.3 (17.9)		74.3 (13.7)		74 (14.8)		58.4 (16.7)		<0.001
Male sex		651	49.1 % (46.4–51.8)	513	48.5 % (45.5–51.5)	83	58.9 %	110	40.4 %	114	55.6 %	9	64.3 %	197	46.4 %	84	57.5 %	22	50 %	0.54
Premorbid medication																				
Antihypertensive treatment		694	52.3 % (49.6–55)	585	55.3 % (52.3–58.3)	84	59.6 %	172	63.2 %	120	58.5 %	0		209	49.2 %	74	50.7 %	13	29.5 %	0.0086
Antiplatelet agent		327	24.7 % (22.3–27)	276	26.1 % (23.5–28.8)	48	34 %	75	27.6 %	54	26.3 %	0		99	23.3 %	37	25.3 %	3	6.8 %	0.0256
Anticoagulant		99	7.5 % (6.1–8.9)	76	7.2 % (5.6–8.7)	4	2.8 %	66	24.3 %	0		0		6	1.4 %	18	12.3 %	3	6.8 %	0.0802
Lipid-lowering drug		70	5.3 % (4.1–6.5)	62	5.9 % (4.4–7.3)	10	7.1 %	10	3.7 %	14	6.8 %	0		28	6.6 %	4	2.7 %	1	2.3 %	0.3287
Premorbid risk factor																				
Hypertension	975		73.5 % (71.2–75.9)	802	75.9 % (73.3–78.5)	105	74.5 %	212	77.9 %	182	88.8 %	3	21.4 %	300	70.6 %	120	82.2 %	22	50 %	0.0057
Diabetes mellitus	225		17 % (14.9–19)	200	18.9 % (16.6–21.3)	33	23.4 %	44	16.2 %	53	25.9 %	0		70	16.5 %	17	11.6 %	3	6.8 %	0.0087
Hypercholesterolemia	375		28.3 % (25.9–30.7)	340	32.2 % (29.3–35)	48	34 %	59	21.7 %	85	41.5 %	2	14.3 %	146	34.4 %	22	15.1 %	3	6.8 %	<0.0001
Previous TIA	110		8.3 % (6.8–9.8)	99	9.4 % (7.6–11.1)	15	10.6 %	21	7.7 %	26	12.7 %	1	7.1 %	36	8.5 %	5	3.4 %	0		0.0284
Know previous atrial fibrillation	266		20.1 % (17.9–22.2)	235	22.2 % (19.7–24.7)	10	7.1 %	206	75.7 %	1	0.5 %	0		18	4.2 %	23	15.8 %	2	4.5 %	0.0030
Previous myocardial infarction	152		11.5 % (9.7–13.2)	134	12.7 % (10.7–14.7)	29	20.6 %	37	13.6 %	19	9.3 %	0		49	11.5 %	13	8.9 %	2	4.5 %	
Current smoking	191		14.4 % (12.5–16.3)	167	15.8 % (13.6–18)	33	23.4 %	24	8.8 %	46	22.4 %	1	7.1 %	66	15.5 %	12	8.2 %	4	9.1 %	0.0889
Heavy drinker	59		4.4 % (3.3–5.6)	52	4.9 % (3.6–6.2)	10	7.1 %	9	3.3 %	20	9.8 %	0		13	3.1 %	6	4.1 %	0		0.2336
BMI >30 (kg/m^2^)	87		6.6 % (5.2–7.9)	77	7.3 % (5.7–8.9)	6	4.3 %	18	6.6 %	21	10.2 %	0		32	7.5 %	5	3.4 %	1	2.3 %	0.2246

* Statistical difference in the mean age was tested using analysis of variance; all other comparisons were tested by χ^2^ test

The ischaemic stroke subtypes differed by age (older for embolic stroke and younger for other causes of stroke, *p* < 0.001); sex (highest female predominance in embolic, *p* < 0.002) (highest male frequency in atherothrombotic stroke, *p* = 0.01); previous atrial fibrillation (highest frequency in embolic stroke, *p* < 0.0001); previous myocardial infarction (highest frequency in atherothrombotic stroke, *p* = 0.04); history of smoking (highest frequency in atherothrombotic stroke, *p* = 0.04); history of hypertension and hypercholesterolaemia (highest frequency in lacunar stroke, *p* = 0.003).

The case-fatality of first-ever stroke was 9.5 % at 7 day, 16.1 % at 28 day, and 29.9 % at 1 year (Table 5). Case-fatality was higher for primary intracerebral haemorrhage, subarachnoid haemorrhage and stroke of undetermined type than for ischaemic stroke. Case-fatality was higher in ischaemic stroke patients with atrial fibrillation than for the overall cohort. Case-fatality at 1 year after ischaemic stroke was 46 % in women with atrial fibrillation compared with 20 % in women without atrial fibrillation (*p* < 0.0001), and 39 % in men with atrial fibrillation compared with 20 % in men without atrial fibrillation (*p* < 0.0001). In patients with ischaemic stroke the case-fatality was higher for embolic stroke subtype than lacunar (*p* < 0.0001).

**Table 5:** Case–fatality at 7 days, 28 days and 1 year in CARe Aosta Valley, Italy 2004–2008

	Case–fatality					
	Dead at 7 days		Dead at 28 days		Dead at 1 year	
	*n*	Rate (95 % CI)	*n*	Rate (95 % CI)	*n*	Rate (95 % CI)
First-ever stroke (*n* = 1,326)	126	9.5 % (7.9–11.1)	214	16.1 % (14.2–18.1)	396	29.9 % (27.4–32.3)
Sex						
Male	55	8.4 % (6.3–10.6)	97	14.9 % (12.2–17.6)	187	28.7 % (25.2–32.2)
Female	71	10.5 % (8.2–12.8)	117	17.3 % (14.5–20.2)	209	31 % (27.5–34.4)
*p*	0.2823		0.3423		0.5505	
Age group, years						
0–64	14	6.1 % (3–9.2)	20	8.7 % (5.1–12.3)	30	13 % (8.7–17.4)
65–84	59	7.9 % (6–9.8)	99	13.3 % (10.8–15.7)	193	25.8 % (22.7–29)
≥85	53	15.2 % (11.4–19)	95	27.2 % (22.5–31.9)	173	49.6 % (44.3–54.8)
*p*	0.0006		<0.0001		<0.0001	
Ischaemic stroke (*n* = 1,057)	49	4.6 % (3.4–5.9)	114	10.8 % (8.9–12.7)	267	25.3 % (22.6–27.9)
Atherothrombotic (*n* = 153)	1	0.7 % (0–1.9)	10	6.5 % (2.6–10.5)	28	18.3 % (12.2–24.4)
Embolic (*n* = 266)	25	9.4 % (5.9–12.9)	50	18.8 % (14.1–23.5)	113	42.5 % (36.5–48.4)
Lacunar (*n* = 210)	1	0.5 % (0–1.4)	2	1 % (0–2.3)	13	6.2 % (2.9–9.4)
Other (*n* = 16)	0		1	6.2 % (0–18.1)	1	6.2 % (0–18.1)
Undefined (*n* = 412)	22	5.3 % (3.2–7.5)	51	12.4 % (9.2–15.6)	112	27.2 % (22.9–31.5)
*p*	<0.0001		<0.0001		<0.0001	
Primary intracerebral haemorrhage (*n* = 146)	33	22.6 % (15.8–29.4)	48	32.9 % (25.3–40.5)	70	47.9 % (39.8–56)
Subarachnoid haemorrhage (*n* = 44)	7	15.9 % (5.1–26.7)	12	27.3 % (14.1–40.4)	14	31.8 % (18.1–45.6)
Stroke of undetermined type (*n* = 79)	37	46.8 % (35.8–57.8)	40	50.6 % (39.6–61.7)	45	57 % (46–67.9)
*p*	<0.0001		<0.0001		<0.0001	

Statistical differences were tested by χ^2^ test

Follow-up data were recorded 3.76 years (95 % CI 3.6–3.9) after the first-ever stroke for 1,269 incident cases (595 patients died), but 57 were lost to follow-up. Among these survivors 69 % were functionally independent. There were significant gender differences (women presented a worse prognosis, *p* < 0.001), age differences (elderly patients had the worst prognosis, *p* = 0.0001) and ischaemic stroke subtypes differences (embolic stroke had the worst prognosis, *p* = 0.009) (Table 6).

**Table 6:** mRS at 3.7 years in CARe Aosta Valley, Italy 2004–2008

	mRS 0–2		mRS 3–5	
	*n*	Rate (95 % CI)	*n*	Rate (95 % CI)
First-ever stroke	530	69.7 % (66.5–73	230	30.3 % (27–33.5)
Sex				
Male	291	75.8 % (71.5–80.1)	93	24.2 % (19.9–28.5
Female	239	63.6. % (58.7–68.4)	137	36.4 % (31.6–41.3)
*p*	<0.001		<0.001	
Age group, years				
0–64	163	86.2 % (81.3–91.2)	26	13.8 % (8.8–18.7)
65–84	327	70.6 % (66.5–74.8)	136	29.4 % (25.2–33.5)
≥85	40	37 % (27.9–46.1)	68	63 % (53.9–72.1)
*p*	0.0003		<0.0001	
Ischaemic stroke	455	70.4 % (66.9–74)	191	29.6 % (26–33.1)
Atherothrombotic	62	63.3 % (53.7–72.8)	36	36.7 % (27.2–46.3)
Embolic	69	56.6 % (47.8–65.4)	53	43.4 % (34.6–52.2)
Lacunar	127	75.1 % (68.6–81.7)	42	24.9 % (18.3–31.4)
Other	14	100 % (100–100)	0	
Undefined	183	75.3 % (69.9–80.7)	60	24.7 % (19.3–30.1)
*p*	0.3784		0.0090	
Primary intracerebral haemorrhage	35	58.3 % (45.9–70.8)	25	41.7 % (29.2–54.1)
Subarachnoid haemorrhage	19	73.1 % (56–90.1)	7	26.9 % (9.9–44)
Stroke of undetermined type	21	75 % (59–91)	7	25 % (9–41)
*p*	0.8445		0.5358	

Statistical differences were tested by χ^2^ test

**Fig. 1:**
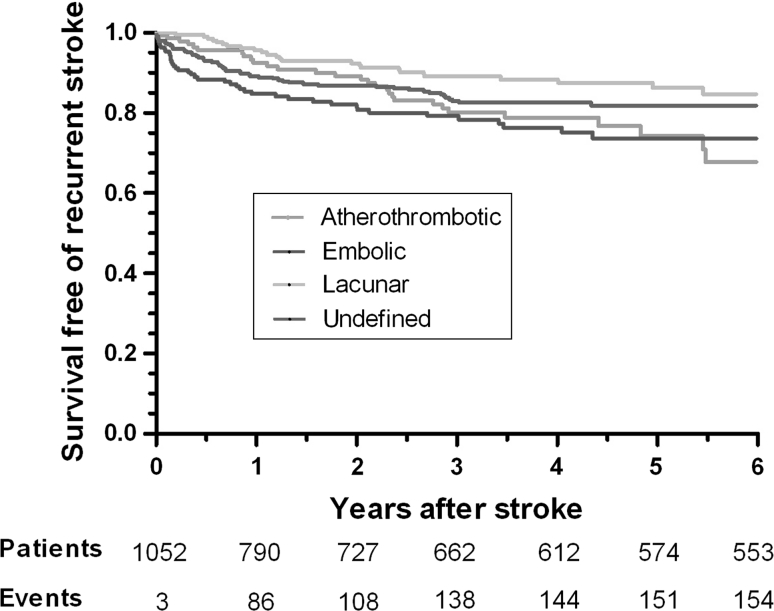
Observed percentage surviving free of recurrent stroke after first-ever stroke in CARe, with common ischaemic subtypes

Twenty-eight percent of ischaemic stroke followed up patients (154/553) had a recurrent stroke, 64 % (109/154) of these recurrent strokes were of the same subtype as the incident stroke and 12 % (18/154) of these were fatal. Eleven per cent of patients (86/790) had recurrent stroke within 1 year, 20 % (30/153) of whom had embolic stroke as the first stroke subtype (*p* < 0.002). Figure 1 presents the Kaplan–Meier estimates of recurrent stroke for the different subtypes. Recurrence rate was 3.4 % at 1 year for patients with primary intracerebral haemorrhage; no events were recorded in patients with subarachnoid haemorrhage.

## Discussion

**Fig. 2:**
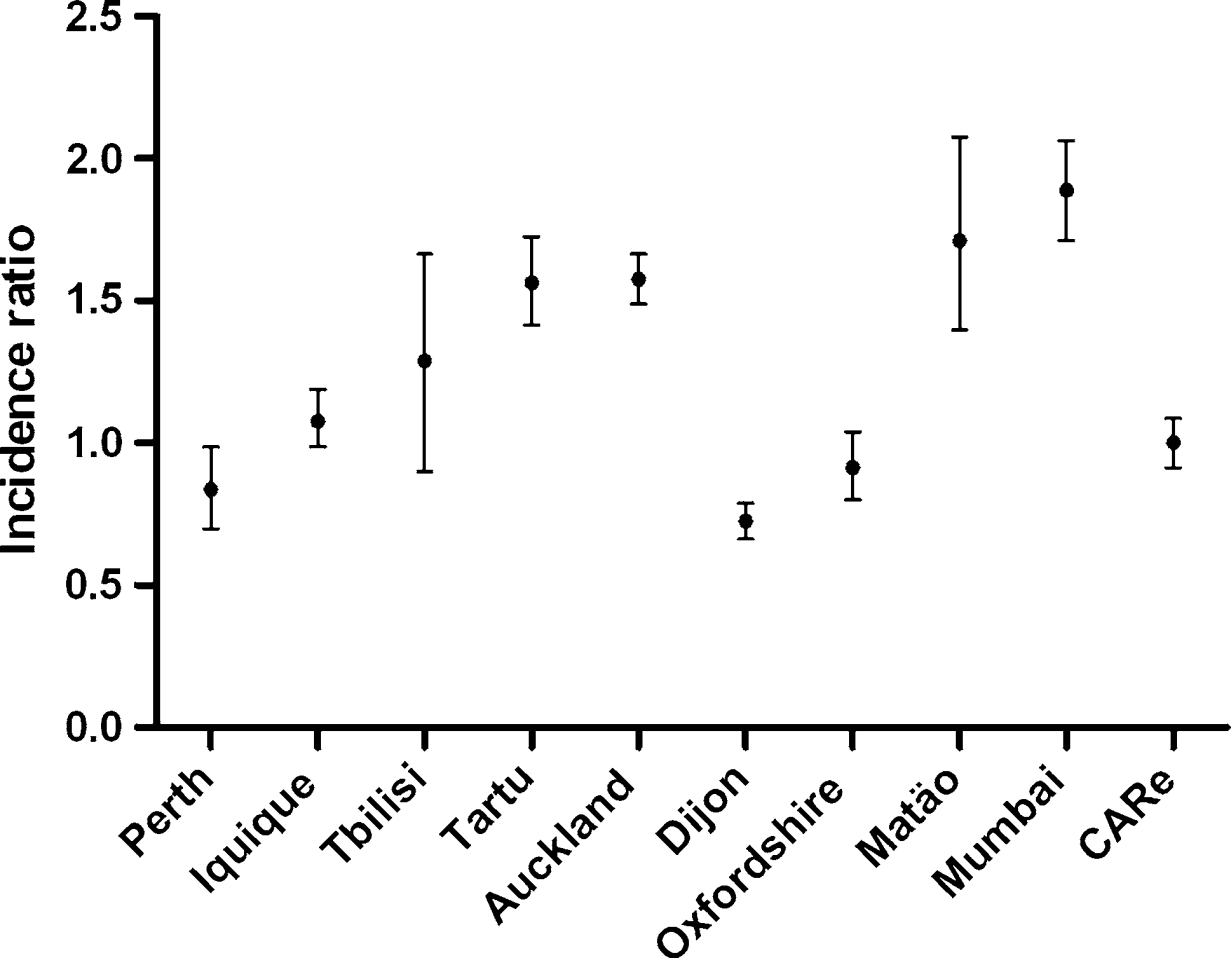
Rate ratios of age-adjusted (age-adjusted to world population) incidence rates for first-ever stoke in population-based studies in the last decade. Reference group is CARe. *Circles* are means and *bar* are 95 % CI

The present study shows a lower incidence rate of first-ever stroke, adjusted to the world population, when compared to studies conducted in the last decade [22], except for the Oxfordshire study [23], the Perth study [24], and the Dijon study [25], Fig. 2. These data, however, are rarely totally comparable due to the different methodology and the specific Health System of each country. In the last two decades the Italian registries have reported variable crude and age-adjusted stroke incidence rates [22, 26, 27]. This variability may be due to different diagnostic criteria, methodological biases and local population age.

The authors followed the recruitment criteria and stroke classification criteria published by Sudlow and Warlow [15], and by Feigin [28]. Complete case ascertainment in our study is assured by four principal factors. First, in Italy, the “Sistema Sanitario Nazionale” is the only medical provider of stroke services, and the access to medical care is free of charge for people of all ages. Second, the specific geographical location of AV (mountainous) and the fact that only one hospital with a stroke unit serves the entire area means that the vast majority of cases are referred and are followed up there. The only hospital in the boundary of the AV is situated in Ivrea (10 km from the border), and this hospital was contacted every year till 2009, to check on data on AV residents, admitted with a diagnosis of stroke. Third, a large number of cases are referred to the outpatient clinic by GPs, these patients are then diagnosed as TIA; thus very few cases of very mild ischaemic stroke or TIA could have been missed. Fourth, GPs identified and referred the remaining 3 % of cases to the study group, who are AV residents but were taken ill and hospitalised whilst in another area. However, our finding of 7 % cases of outpatient strokes was similar to that of other European population studies [6, 10].

Data of different studies are not fully comparable; however, we tried to compare our studies with a previous study on the AV. In particular in the 1989–1997 interval an increase in stroke incidence was reported [29]: ageing of the population and better cases identification could explain the higher incidence rate. In a previous study (2004–2005) we reported a major reduction in the incidence of stroke (29 %) between 1989 and 2004–2005 [12]. The decrement of cases might be due to the prevention campaigns started in-between those years. The observed reduction in stroke incidence is therefore likely to be related to better control of vascular risk factors. The analysis of risk factors in our study shows a higher proportion of patients with hypertension, atrial fibrillation and hypercholesterolaemia compared to other studies [7, 9, 23]. Hypertension was significantly prevalent for primary intracerebral haemorrhage. Atrial fibrillation was an important risk factor, and ischaemic stroke patients have a higher mortality risk than patients in sinus rhythm. Embolic stroke was particularly frequent in the elderly people and in particular female patients. It has to be noted that only one-third of embolic stroke patients with atrial fibrillation were treated with anticoagulants before the stroke event, and this cannot be explained by contraindication to anticoagulant therapy alone. Previous reports, in Italy, have pointed out that atrial fibrillation is under-recognised [30], and the underuse of anticoagulant therapy is still common [31]. These findings showed still insufficient stroke prevention measures in our study population. Significant differences were found among stroke subtypes, atherothrombotic stroke cases were mainly elderly male smokers with a history of a previous myocardial infarction, and lacunar stroke cases had more frequently hypertension and hypercholesterolemia. These findings were different from those of the Oxfordshire [23] and the Rochester studies [9]. Although these differences in risk factors may be due to differences in age, sex, race among study subjects, we believe methodological differences between studies are a more likely explanation. Ischaemic stroke of undetermined cause was the most common subtype. Difficulties in classifying patients according to the TOAST classification are more frequent in patients in a community-based setting rather than in a hospital setting.

The overall 28-day case-fatality for first-ever stroke was lesser than that found in most studies [22, 25–27], and was similar for men and women. The increased hospitalisation rate in our region could have contributed to the decline in case-fatality [12, 27]. Patients with primary intracerebral haemorrhage or subarachnoid haemorrhage had an almost twofold probability of dying compared to patients with ischaemic stroke, moreover mortality increased significantly in elderly patients. Few studies gave results for 1-year mortality by age, sex, pathological types [5, 6, 10, 26] and ischaemic subtypes [11]. Case-fatality at 1 year for ischaemic stroke and primary intracerebral haemorrhage were lesser in our area than in Aquila [26], this may be explained by the differing age of patients in the two study populations. Age is one of the most important predictors of unfavourable stroke outcomes, a significant correlation was found between age and stroke mortality. After 1 year, the probability of survival after stroke was higher for ischaemic stroke than for other stroke types. Embolic stroke was associated with highest mortality, and lacunar stroke had the lowest mortality.

The importance of our study resides in the reports of outcomes for stroke types and the disability for each ischaemic stroke subtypes among all hospitalised and non-hospitalised patients in a community. At follow-up, 69 % of patients were able to independently carry out daily living activities without significant differences among stroke types. Patients functionally independent at 1 year were 65 and 68.9 % in the Oxfordshire [23] and Arcadia [10] study, respectively. Differences were found in ischaemic stroke subtypes: lacunar stroke had the best functional outcomes, with 75 % having minimal or no impairment after the stroke, and embolic stroke the worst functional outcome, 43 % of patients were dependent. The quantification of risk of recurrence associated with ischaemic stroke due to embolic stroke differs from what found in a previous study [1]. More than 20 % of patients with this subtype had a recurrent stroke within 1 year from the first stroke, which means that further improvement in risk factor reduction in high-risk patients with embolic stroke is necessary, because patients who survive a stroke associated with atrial fibrillation may have increased frequency of recurrence and more severe functional deficits. Once again rates of dependency increased with age, significantly in the elderly group. Female patients have greater difficulty than male patients in recovering from a disabled state after stroke. The precise reasons for gender differences in post-stroke functional status remain unclear. Long-term functional outcomes might be influenced by various factors. Our data suggest that differences in mean age between genders, differing distributions of stroke subtypes may influence gender differences in functional outcomes.

Overall differences were found in the distribution of risk factors and prognosis across the stroke types and ischaemic stroke subtypes. Increasing age, female gender and embolic stroke subtypes were associated with an adverse outcome. Prevention efforts should be directed at decreasing this number of new strokes by a better control of risk factors, particularly atrial fibrillation, in elderly patients.
